# 4,4′-Bipyridine–2-meth­oxy­benzoic acid (1/2)

**DOI:** 10.1107/S1600536812003194

**Published:** 2012-02-04

**Authors:** Xiao-Yan Qian, Feng-Zhi Liu

**Affiliations:** a Suzhou Industrial Park Centers for Disease Control and Prevention, Institute of Health Inspection and Supervision, 215021 Suzhou, Jiangsu, People’s Republic of China

## Abstract

The asymmetric unit of the title compound, C_10_H_8_N_2_·2C_8_H_8_O_3_, contains two 2-meth­oxy­benzoic acid mol­ecules and one 4,4′-bipyridine mol­ecule. The 4,4′-bipyridine mol­ecule is disordered over two positions in a 1:1 ratio. In the crystal, the 2-meth­oxy­benzoic acid and 4,4′-bipyridine mol­ecules are connected by inter­molecular O—H⋯N hydrogen bonds. The dihedral angle between the carboxy group and its attached ring is 26.823 (2)°.

## Related literature

For the use and related structures of 2-meth­oxy­benzoic acid in coordination chemistry, see: Vollano *et al.* (1984 ▶); Smith *et al.* (1986 ▶); Li (2005 ▶); Andrews *et al.* (2006 ▶); Ren *et al.* (2006 ▶); Zhao *et al.* (2008 ▶); Sharma *et al.* (2009 ▶).
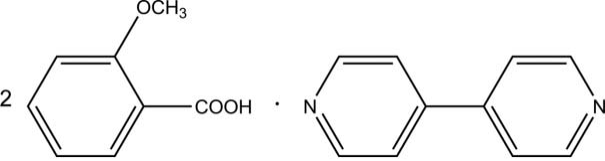


## Experimental

### 

#### Crystal data

C_10_H_8_N_2_·2C_8_H_8_O_3_
*M* *_r_* = 460.47
Monoclinic, 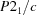

*a* = 7.7090 (15) Å
*b* = 25.620 (5) Å
*c* = 6.3624 (13) Å
β = 112.08 (3)°
*V* = 1164.4 (4) Å^3^
*Z* = 2
Mo *K*α radiation
μ = 0.09 mm^−1^
*T* = 298 K
0.30 × 0.28 × 0.25 mm

#### Data collection

Bruker SMART APEXII CCD diffractometer
Absorption correction: multi-scan (*SADABS*; Bruker, 2005 ▶) *T*_min_ = 0.972, *T*_max_ = 0.977
5991 measured reflections
2060 independent reflections
1540 reflections with *I* > 2σ(*I*)
*R*_int_ = 0.088

#### Refinement

*R*[*F* ^2^ > 2σ(*F* ^2^)] = 0.052
*wR*(*F* ^2^) = 0.154
*S* = 1.06
2060 reflections
172 parameters
9 restraints
H-atom parameters constrained
Δρ_max_ = 0.15 e Å^−3^
Δρ_min_ = −0.16 e Å^−3^

### 

Data collection: *APEX2* (Bruker, 2005 ▶); cell refinement: *APEX2*; data reduction: *SAINT* (Bruker, 2005 ▶); program(s) used to solve structure: *SHELXTL* (Sheldrick, 2008 ▶); program(s) used to refine structure: *SHELXTL*; molecular graphics: *SHELXTL*; software used to prepare material for publication: *SHELXTL*.

## Supplementary Material

Crystal structure: contains datablock(s) global, I. DOI: 10.1107/S1600536812003194/ds2171sup1.cif

Structure factors: contains datablock(s) I. DOI: 10.1107/S1600536812003194/ds2171Isup2.hkl

Supplementary material file. DOI: 10.1107/S1600536812003194/ds2171Isup3.cml

Additional supplementary materials:  crystallographic information; 3D view; checkCIF report

## Figures and Tables

**Table 1:** Hydrogen-bond geometry (Å, °)

*D*—H⋯*A*	*D*—H	H⋯*A*	*D*⋯*A*	*D*—H⋯*A*
O1—H1⋯N1^i^	0.82	1.85	2.673 (2)	177

Symmetry code: (i) 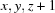.

**Fig. 1.**
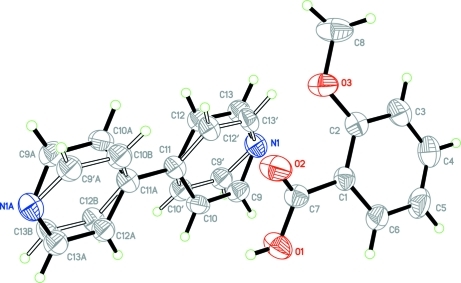
View of the title compound showing the atomic labeling and 30% probability displacement ellipsoids.

**Fig. 2.**
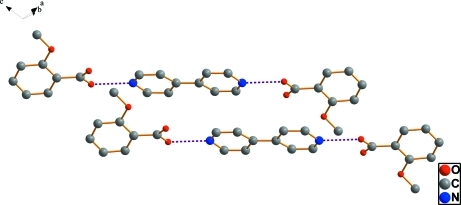
The O—H···N hydrogen bonds of (I). All H atoms have been omitted for clarity. The dashed lines indicate the O—H···N hydrogen bonds.

## supplementary crystallographic information

### Comment

Bipyridine is a well known molecule often used as a linker in polymeric coordination complexes. 2-Methoxybenzoic acid is also sometimes used as a common ligand in coordination polymers (Vollano *et al.*, 1984; Smith *et al.*, 1986; Li, 2005; Andrews *et al.*, 2006; Ren *et al.*, 2006; Zhao *et al.*, 2008; Sharma *et al.*, 2009.). The title compound, (I), is a 1:2 cocrystal of the aforementioned linkers. Herewith we present its crystal structure. The asymmetric unit of the title compound (Fig. 1) contains two 2-methoxybenzoic acid molecules and one 4,4'-bipyridine molecule. The dihedral angle of carboxy group to its ring is 26.823 (2)°. The 4,4'-bipyridine molecule is disordered over two positions in a 1:1 ratio. In the crystal structure, the 2-methoxybenzoic acid and 4,4'-bipyridine are held together by intermolecular O—H···N hydrogen bonds.

### Experimental

An ethanol solution (20 ml) of 2-methoxybenzoic acid (0.1 mmol) and 4,4'-bipyridine (0.1 mmol) was heated at 333 K for 2 h. Then the mixture was cooled to room temperature. After two weeks colorless crystals were obtained that were suitable for X-ray diffraction study.

### Refinement

Four C atoms of bipyridyl group are disordered over two sites. The occupancy factors refined to 0.761 (2) and 0.239 (2). H atoms were positioned geometrically and refined as riding groups, with O—H = 0.82 Å, C_aromatic_—H = 0.93 Å and C_methyl_—H = 0.96 Å and with *U*_iso_(H) = 1.2*U*_eq_(C_methyl_,O) and *U*_iso_(H) = 1.5*U*_eq_(_aromatic_), respectively.

### Crystal data

**Table:**

C_10_H_8_N_2_·2C_8_H_8_O_3_	*F*(000) = 484
*M**_r_* = 460.47	*D*_x_ = 1.313 Mg m^−^^3^
Monoclinic, *P*2_1_/*c*	Mo *K*α radiation, λ = 0.71073 Å
Hall symbol: -P 2ybc	Cell parameters from 2567 reflections
*a* = 7.7090 (15) Å	θ = 2.4–23.4°
*b* = 25.620 (5) Å	µ = 0.09 mm^−^^1^
*c* = 6.3624 (13) Å	*T* = 298 K
β = 112.08 (3)°	Block, colourless
*V* = 1164.4 (4) Å^3^	0.30 × 0.28 × 0.25 mm
*Z* = 2	

### Data collection

**Table:**

Bruker SMART APEXII CCD diffractometer	2060 independent reflections
Radiation source: fine-focus sealed tube	1540 reflections with *I* > 2σ(*I*)
Graphite monochromator	*R*_int_ = 0.088
ω scans	θ_max_ = 25.1°, θ_min_ = 2.9°
Absorption correction: multi-scan (*SADABS*; Bruker, 2005)	*h* = −6→9
*T*_min_ = 0.972, *T*_max_ = 0.977	*k* = −30→29
5991 measured reflections	*l* = −7→7

### Refinement

**Table:**

Refinement on *F*^2^	Primary atom site location: structure-invariant direct methods
Least-squares matrix: full	Secondary atom site location: difference Fourier map
*R*[*F*^2^ > 2σ(*F*^2^)] = 0.052	Hydrogen site location: inferred from neighbouring sites
*wR*(*F*^2^) = 0.154	H-atom parameters constrained
*S* = 1.06	*w* = 1/[σ^2^(*F*_o_^2^) + (0.0805*P*)^2^ + 0.1072*P*] where *P* = (*F*_o_^2^ + 2*F*_c_^2^)/3
2060 reflections	(Δ/σ)_max_ < 0.001
172 parameters	Δρ_max_ = 0.15 e Å^−^^3^
9 restraints	Δρ_min_ = −0.16 e Å^−^^3^

### Special details

**Table:**

Geometry. All e.s.d.'s (except the e.s.d. in the dihedral angle between two l.s. planes) are estimated using the full covariance matrix. The cell e.s.d.'s are taken into account individually in the estimation of e.s.d.'s in distances, angles and torsion angles; correlations between e.s.d.'s in cell parameters are only used when they are defined by crystal symmetry. An approximate (isotropic) treatment of cell e.s.d.'s is used for estimating e.s.d.'s involving l.s. planes.
Refinement. Refinement of *F*^2^ against ALL reflections. The weighted *R*-factor *wR* and goodness of fit *S* are based on *F*^2^, conventional *R*-factors *R* are based on *F*, with *F* set to zero for negative *F*^2^. The threshold expression of *F*^2^ > σ(*F*^2^) is used only for calculating *R*-factors(gt) *etc*. and is not relevant to the choice of reflections for refinement. *R*-factors based on *F*^2^ are statistically about twice as large as those based on *F*, and *R*-factors based on ALL data will be even larger.

### Fractional atomic coordinates and isotropic or equivalent isotropic displacement parameters (Å^2^)

**Table:**

	*x*	*y*	*z*	*U*_iso_*/*U*_eq_	Occ. (<1)
O1	0.4229 (2)	0.58341 (6)	0.9083 (3)	0.0771 (5)	
H1	0.3645	0.5715	0.9816	0.116*	
C1	0.4341 (3)	0.65024 (7)	0.6614 (3)	0.0525 (5)	
O2	0.1962 (2)	0.64201 (7)	0.8143 (3)	0.0791 (5)	
C2	0.3393 (3)	0.68089 (7)	0.4708 (3)	0.0562 (5)	
O3	0.1512 (2)	0.68604 (6)	0.4068 (2)	0.0717 (5)	
C3	0.4378 (4)	0.70402 (9)	0.3514 (4)	0.0751 (7)	
H3	0.3747	0.7242	0.2241	0.090*	
C4	0.6271 (4)	0.69715 (11)	0.4209 (5)	0.0877 (8)	
H4	0.6916	0.7131	0.3408	0.105*	
C5	0.7227 (3)	0.66730 (11)	0.6053 (6)	0.0875 (8)	
H5	0.8513	0.6627	0.6505	0.105*	
C6	0.6260 (3)	0.64391 (9)	0.7248 (4)	0.0702 (6)	
H6	0.6912	0.6235	0.8505	0.084*	
C7	0.3370 (3)	0.62529 (8)	0.7980 (3)	0.0545 (5)	
C8	0.0540 (4)	0.71555 (13)	0.2045 (4)	0.0945 (9)	
H8A	0.0982	0.7509	0.2256	0.142*	
H8B	−0.0779	0.7151	0.1734	0.142*	
H8C	0.0767	0.7004	0.0793	0.142*	
N1	0.2439 (3)	0.54252 (7)	0.1570 (3)	0.0661 (5)	
C11	0.0512 (3)	0.50894 (7)	0.4279 (3)	0.0556 (5)	
C9	0.3203 (7)	0.5048 (2)	0.3112 (9)	0.0635 (12)	0.50
H9	0.4362	0.4910	0.3277	0.076*	0.50
C10	0.2284 (7)	0.48633 (19)	0.4455 (9)	0.0616 (11)	0.50
H10	0.2804	0.4593	0.5474	0.074*	0.50
C12	−0.0112 (8)	0.5490 (2)	0.2734 (9)	0.0581 (18)*	0.50
H12	−0.1225	0.5658	0.2568	0.070*	0.50
C13	0.0872 (8)	0.5649 (3)	0.1426 (10)	0.063 (2)*	0.50
H13	0.0406	0.5923	0.0411	0.075*	0.50
C9'	0.2515 (7)	0.49348 (19)	0.2343 (9)	0.0625 (16)*	0.50
H9'	0.3280	0.4698	0.1990	0.075*	0.50
C10'	0.1531 (8)	0.4757 (2)	0.3634 (9)	0.0645 (16)*	0.50
H10'	0.1587	0.4407	0.4043	0.077*	0.50
C12'	0.0289 (8)	0.55892 (18)	0.3415 (9)	0.0554 (13)	0.50
H12'	−0.0516	0.5823	0.3710	0.066*	0.50
C13'	0.1266 (8)	0.57342 (19)	0.2127 (10)	0.0599 (14)	0.50
H13'	0.1112	0.6075	0.1585	0.072*	0.50

### Atomic displacement parameters (Å^2^)

**Table:**

	*U*^11^	*U*^22^	*U*^33^	*U*^12^	*U*^13^	*U*^23^
O1	0.0796 (11)	0.0708 (10)	0.0986 (12)	0.0224 (8)	0.0536 (9)	0.0295 (9)
C1	0.0532 (11)	0.0469 (10)	0.0603 (11)	−0.0023 (8)	0.0247 (9)	−0.0035 (9)
O2	0.0730 (10)	0.0971 (12)	0.0823 (11)	0.0283 (8)	0.0465 (8)	0.0295 (9)
C2	0.0632 (12)	0.0525 (10)	0.0581 (11)	0.0013 (9)	0.0287 (9)	−0.0044 (9)
O3	0.0667 (9)	0.0895 (11)	0.0631 (9)	0.0218 (7)	0.0292 (7)	0.0171 (8)
C3	0.0904 (17)	0.0689 (14)	0.0751 (14)	−0.0069 (12)	0.0413 (13)	0.0071 (11)
C4	0.0878 (19)	0.0886 (17)	0.103 (2)	−0.0228 (14)	0.0550 (16)	0.0040 (15)
C5	0.0574 (13)	0.0915 (18)	0.121 (2)	−0.0166 (12)	0.0426 (14)	−0.0042 (17)
C6	0.0533 (12)	0.0671 (13)	0.0882 (15)	−0.0044 (10)	0.0244 (11)	0.0036 (11)
C7	0.0532 (11)	0.0563 (11)	0.0562 (11)	0.0063 (9)	0.0231 (9)	0.0015 (9)
C8	0.0963 (18)	0.126 (2)	0.0663 (14)	0.0422 (16)	0.0362 (13)	0.0282 (15)
N1	0.0785 (12)	0.0605 (10)	0.0678 (11)	−0.0008 (9)	0.0373 (9)	0.0010 (9)
C11	0.0683 (12)	0.0470 (10)	0.0550 (11)	−0.0013 (9)	0.0272 (9)	−0.0049 (8)
C9	0.054 (3)	0.076 (3)	0.060 (3)	0.003 (2)	0.022 (2)	−0.001 (2)
C10	0.059 (3)	0.067 (3)	0.059 (3)	0.013 (2)	0.022 (2)	0.018 (2)
C12'	0.072 (3)	0.042 (2)	0.055 (3)	−0.005 (2)	0.028 (2)	−0.010 (2)
C13'	0.083 (3)	0.043 (2)	0.054 (3)	−0.012 (2)	0.026 (3)	−0.002 (2)

### Geometric parameters (Å, º)

**Table:**

O1—C7	1.316 (2)	N1—C9'	1.343 (5)
O1—H1	0.8200	N1—C9	1.345 (4)
C1—C6	1.389 (3)	N1—C13'	1.346 (5)
C1—C2	1.398 (3)	C11—C10'	1.325 (6)
C1—C7	1.488 (3)	C11—C12	1.377 (6)
O2—C7	1.207 (2)	C11—C12'	1.378 (5)
C2—O3	1.357 (2)	C11—C10	1.448 (4)
C2—C3	1.392 (3)	C11—C11^i^	1.490 (4)
O3—C8	1.437 (3)	C9—C10	1.382 (5)
C3—C4	1.368 (3)	C9—H9	0.9300
C3—H3	0.9300	C10—H10	0.9300
C4—C5	1.362 (4)	C12—C13	1.381 (7)
C4—H4	0.9300	C12—H12	0.9300
C5—C6	1.385 (4)	C13—H13	0.9300
C5—H5	0.9300	C9'—C10'	1.388 (6)
C6—H6	0.9300	C9'—H9'	0.9300
C8—H8A	0.9600	C10'—H10'	0.9300
C8—H8B	0.9600	C12'—C13'	1.357 (6)
C8—H8C	0.9600	C12'—H12'	0.9300
N1—C13	1.309 (6)	C13'—H13'	0.9300
			
C7—O1—H1	109.5	C10'—C11—C12	110.4 (4)
C6—C1—C2	118.00 (18)	C10'—C11—C12'	118.3 (3)
C6—C1—C7	119.65 (19)	C12—C11—C10	114.8 (3)
C2—C1—C7	122.34 (17)	C12'—C11—C10	112.0 (3)
O3—C2—C3	122.4 (2)	C10'—C11—C11^i^	120.4 (3)
O3—C2—C1	117.74 (17)	C12—C11—C11^i^	123.0 (3)
C3—C2—C1	119.9 (2)	C12'—C11—C11^i^	121.0 (3)
C2—O3—C8	117.29 (17)	C10—C11—C11^i^	122.1 (3)
C4—C3—C2	120.2 (2)	N1—C9—C10	120.8 (4)
C4—C3—H3	119.9	N1—C9—H9	119.6
C2—C3—H3	119.9	C10—C9—H9	119.6
C5—C4—C3	121.1 (2)	C9—C10—C11	120.2 (4)
C5—C4—H4	119.4	C9—C10—H10	119.9
C3—C4—H4	119.4	C11—C10—H10	119.9
C4—C5—C6	119.1 (2)	C11—C12—C13	121.7 (5)
C4—C5—H5	120.4	C11—C12—H12	119.1
C6—C5—H5	120.4	C13—C12—H12	119.1
C5—C6—C1	121.6 (2)	N1—C13—C12	122.0 (6)
C5—C6—H6	119.2	N1—C13—H13	119.0
C1—C6—H6	119.2	C12—C13—H13	119.0
O2—C7—O1	122.15 (18)	N1—C9'—C10'	124.5 (5)
O2—C7—C1	124.29 (18)	N1—C9'—H9'	117.7
O1—C7—C1	113.52 (17)	C10'—C9'—H9'	117.7
O3—C8—H8A	109.5	C11—C10'—C9'	119.4 (5)
O3—C8—H8B	109.5	C11—C10'—H10'	120.3
H8A—C8—H8B	109.5	C9'—C10'—H10'	120.3
O3—C8—H8C	109.5	C13'—C12'—C11	118.7 (4)
H8A—C8—H8C	109.5	C13'—C12'—H12'	120.6
H8B—C8—H8C	109.5	C11—C12'—H12'	120.6
C13—N1—C9'	110.4 (4)	N1—C13'—C12'	125.4 (4)
C13—N1—C9	120.3 (4)	N1—C13'—H13'	117.3
C9'—N1—C13'	113.3 (3)	C12'—C13'—H13'	117.3
C9—N1—C13'	112.6 (3)		
			
C6—C1—C2—O3	178.65 (17)	C10'—C11—C12—C13	−28.0 (6)
C7—C1—C2—O3	−2.3 (3)	C12'—C11—C12—C13	88.6 (12)
C6—C1—C2—C3	−0.2 (3)	C10—C11—C12—C13	1.7 (7)
C7—C1—C2—C3	178.92 (18)	C11^i^—C11—C12—C13	179.7 (4)
C3—C2—O3—C8	1.7 (3)	C9'—N1—C13—C12	24.7 (7)
C1—C2—O3—C8	−177.1 (2)	C9—N1—C13—C12	−3.9 (8)
O3—C2—C3—C4	−179.2 (2)	C13'—N1—C13—C12	−77.4 (12)
C1—C2—C3—C4	−0.4 (3)	C11—C12—C13—N1	0.5 (9)
C2—C3—C4—C5	0.7 (4)	C13—N1—C9'—C10'	−23.9 (7)
C3—C4—C5—C6	−0.5 (4)	C9—N1—C9'—C10'	93.1 (9)
C4—C5—C6—C1	−0.1 (4)	C13'—N1—C9'—C10'	−1.4 (7)
C2—C1—C6—C5	0.4 (3)	C12—C11—C10'—C9'	28.7 (6)
C7—C1—C6—C5	−178.7 (2)	C12'—C11—C10'—C9'	7.6 (7)
C6—C1—C7—O2	151.7 (2)	C10—C11—C10'—C9'	−76.1 (7)
C2—C1—C7—O2	−27.4 (3)	C11^i^—C11—C10'—C9'	−178.2 (4)
C6—C1—C7—O1	−26.2 (3)	N1—C9'—C10'—C11	−3.8 (8)
C2—C1—C7—O1	154.75 (18)	C10'—C11—C12'—C13'	−6.4 (7)
C13—N1—C9—C10	4.8 (8)	C12—C11—C12'—C13'	−78.6 (12)
C9'—N1—C9—C10	−70.7 (8)	C10—C11—C12'—C13'	23.5 (7)
C13'—N1—C9—C10	26.7 (7)	C11^i^—C11—C12'—C13'	179.4 (5)
N1—C9—C10—C11	−2.5 (9)	C13—N1—C13'—C12'	89.0 (13)
C10'—C11—C10—C9	86.1 (8)	C9'—N1—C13'—C12'	2.6 (8)
C12—C11—C10—C9	−0.7 (7)	C9—N1—C13'—C12'	−27.3 (8)
C12'—C11—C10—C9	−23.2 (7)	C11—C12'—C13'—N1	1.2 (10)
C11^i^—C11—C10—C9	−178.8 (4)		

Symmetry code: (i) −*x*, −*y*+1, −*z*+1.

### Hydrogen-bond geometry (Å, º)

**Table:**

*D*—H···*A*	*D*—H	H···*A*	*D*···*A*	*D*—H···*A*
O1—H1···N1^ii^	0.82	1.85	2.673 (2)	177

Symmetry code: (ii) *x*, *y*, *z*+1.
